# Managing Intractable Symptoms of Parkinson's Disease: A Nonsurgical Approach Employing Infralow Frequency Neuromodulation

**DOI:** 10.3389/fnhum.2022.894781

**Published:** 2022-07-08

**Authors:** Stella B. Legarda, P. Andreas Michas-Martin, Dana McDermott

**Affiliations:** Montage Health Neurology, Monterey, CA, United States

**Keywords:** Parkinson's disease, parkinsonism, uncontrolled symptoms of Parkinson's disease, uncontrolled tremors, freezing gait in Parkinson's disease, infralow frequency neuromodulation in Parkinson's disease, cortical beta suppression, non-pharmacological option in advanced Parkinson's disease

## Introduction

Parkinson's disease (PD) is specific to humans and its prevalence has increased over time (Garcia-Ruiz and Espay, 2017). Clinical features range from hyposmia and constipation early in the disease to cogwheel rigidity, tremors, and bradykinesia (classic triad for clinical diagnosis of PD); and later still postural instability, ataxia, and freezing of gait (FOG) experienced by patients in advanced stages of PD (Jancovic, 2008); these are often refractory to standard medications and even DBS (Lilleeng et al., 2015). Dysphonia, dysphagia, and expressionless faces are other distinctive clinical features. Increasing prevalence is related to longer life expectancy and survival with the disorder; however, changes in lifestyle likely contribute as technological advances occasion a reduction in physical exercise relative to our ancestors. Hunter-gatherers are estimated to have engaged in four times the level of physical activity compared to modern humans, (O'Keefea et al., 2011) and vigorous, even moderate exercise is associated with a >30% reduction in risk of developing PD (Yang et al., 2015). Exercise is known to promote the release of neurotrophic factors in the brain that exert neuroprotective effects (Ahlskog, 2011). The literature on the benefits of clinical neurofeedback in managing PD symptoms has not included the method we employ in our neurology clinic, infralow frequency (<0.01 Hz) brain training (ILF). Our purpose is to contribute our experience using ILF in managing our patients with PD.

## Background

### Parkinson's Disease: Etiopathogenesis

Dopaminergic neurons have an intrinsic vulnerability because of their high metabolic demands, serving amongst other important functions the self-generation of rhythms that underlie the regular and diffuse distribution of dopamine throughout the brain to maintain the basic regulation of locomotion, learning, working memory, cognition, emotion, and behavior. Degeneration of dopaminergic neurons in the pars compacta of substantia nigra (SNc) characterize the neuropathology in PD. High energy demands may lead to calcium overload, resulting in mitochondrial dysfunction and oxidative stress, over time resulting in alpha-synuclein misfolding and aggregation into toxic soluble oligomers leading to progressive pathology in PD (Surmeier et al., 2017). Recent literature emphasizes that the presence of neuroinflammation, particularly reactive astrocytes in SNc, is a consistent feature and provides evidence that PD may develop, at least in part, due to astroglial dysfunction (Booth et al., 2017). Other notable brain regions staining highly for alpha-synuclein in experimental non-human primates include layers III and V of the cerebral cortex and the hippocampus (Yang et al., 2021).

A working “mixed hypothesis” has been proposed that PD begins with a multifocal origin (accounting for early hyposmia and constipation) that relies on trans-synaptic spread for progression to new brain regions accounting for later motor-related manifestations as the disease advances with age (Mou et al., 2020). The high incidence and prevalence of PD in the elderly has motivated the hypothesis of diminishing dopaminergic supply in association with the normal aging process; senescence itself is regarded a background for the development of neurodegenerative diseases like PD, wherein accompanying astroglial changes contribute to disease progression (Yuan et al., 2021). Progressive dysfunctions of astrocytes and microglia lead to a microenvironment deleterious for neuronal survival over time, a more likely scenario since the process of neuronal death is fairly rapid and does not fully explain the gradual neurodegeneration characteristic of clinical PD (Joe et al., 2018). Supporting this thinking is the fact that the majority of PD genes identified to date are usually expressed in astrocytes and microglia (Solano et al., 2008).

A growing emphasis on less neurocentric causal mechanisms of PD supports our experience that ILF brain training is helpful in managing the often medically refractory symptoms in PD. The lowest recordable brain frequencies (<0.01 Hz) are believed to be non-neuronal in origin (Lorincz et al., 2009), dependent on the ATP derived from astrocytes. Astrocytes are critical for synaptic learning (plasticity) (Popov et al., 2021); we postulate that ILF brain training strengthens brain networks involved in neuroplasticity.

### Role of Astrocyte Dysfunction in Pathogenesis of Parkinson's Disease

Many of the genes implicated in the development of PD are expressed in astrocytes, sometimes at levels higher than in neurons (Joe et al., 2018). For example, in postmortem studies of human brain samples expression of PARK7 was shown to be higher in astrocytes than in neurons, and in patients with PD this gene was found to be up-regulated in reactive astrocytes. Alpha-synuclein-positive inclusions have been found in both neurons and astrocytes. Expression of the SNCA gene encoding for alpha-synuclein is low in astrocytes, however the alpha-synuclein from dying neurons is taken up by astrocytes, an attempt to remove and degrade alpha-synuclein to maintain a healthy environment for neuronal survival (Booth et al., 2017). These investigators propose that understanding the role of astrocytes in PD will further our understanding of the disease.

In this paper on PD, we need to mention the evolutionary importance of astrocytes. Morphologically there have been far more prominent evolutionary changes in glia than in neurons when comparing humans and other mammals to earlier species. Compared to rodents, cats, and marsupials humans have a much larger glia:neuron ratio, although our massive whales demonstrate the highest ratio of all mammals. Within the human brain itself there are regional differences; the globus pallidus (GP) has the highest glia:neuron ratio of 160:1 compared to 3.6:1 in gray matter (Verkhratsky et al., 2019).

There is a heterogeneity of function among the astrocytes depending where in the brain they exist. Astrocytes support important network hub functions, and play key roles in homeostatic regulatory functions (Linnerbauer et al., 2020). The homeostatic anti-inflammatory activities of astrocytes and their regulation are coming to be understood (Sanmarco et al., 2021). Neurons are functionally dependent on astrocytes; when any astroglial network falters, the dysfunction and even death of neurons dependent on that network is sure to follow.

### Clinical Approach to Parkinson's Disease

In neurology practice managing the refractory, disabling symptoms and limitations in PD is a humbling challenge. Rational polypharmacy in recent decades has provided some meaningful advances; still there are patients who break through their medication control and seek other methods of management. For some the aggressive surgical route of deep brain stimulation (DBS) is a difficult decision; potential benefits include reduced medication, improvements with rigidity and tremors and control of dyskinesias (Limousine and Foltynie, 2019). Long term reports on DBS are in accord that subthalamic nucleus (STN)-DBS provides greater beneficial effects than globus pallidus internus (GPi)-DBS on symptoms in off periods, allowing for reduction of medications, whereas GPi-DBS has a better effect than STN-DBS on reducing levodopa-induced dyskinesias (Odekerken et al., 2013), and confers relatively less of an impact on cognition (Odekerken et al., 2016). Even then, some patients continue to experience an evolution of their PD symptoms or the adverse effects of stimulation, such as dysarthric speech, swallowing disturbances, FOG and postural instability 5 years or more after an initial good response to DBS (Moro et al., 2010). Cognitive impairments have also been consistently reported to progress over many years (Krack et al., 2003; Gervais-Bernard et al., 2009). Newer model device therapies aim to minimize stimulation-related adverse effects reported with DBS (Paff et al., 2020).

### ILF Brain Training in Parkinson's Disease

Motor features of PD are experienced by patients only after some 50% to 80% of dopaminergic neurons have been lost, suggesting a major reliance on compensatory mechanisms in early disease stages (DeMaagd and Phlip, 2015). Excessive fast beta (13–30 Hz) oscillations in the basal ganglia have been observed in patients with PD (Brown et al., 2001); beta-band oscillations in STN correlate with PD symptoms (Little and Brown, 2012); DBS in the STN suppresses beta-band oscillations (Eusebio et al., 2011); and improvements of PD symptoms were correlated with the attenuation of beta band oscillations during adaptive DBS (Tinkhauser et al., 2017).

In our practice we find a non-pharmacological and non-surgical option in infralow frequency brain training (ILF) to be helpful in managing the distressing motor symptoms in our PD patients. Employing this second-generation form of neurofeedback (NF) allows us to also address cognitive comorbidities common to PD (attention disorders, anxiety, memory changes, loss of executive skills). ILF NF may be a good first step in considering patients for potential DBS therapy. When a high-functioning individual with PD gains benefit from ILF and then over time manifests a re-emergence or persistence of symptoms, we have offered to refer them for DBS. We propose an increased brain resilience is also achieved with ILF, preparing especially our elderly PD patients for a successful surgical outcome after DBS. For PD patients who are not candidates for DBS, we find ILF brain training can also improve FOG and postural instabilities.

The literature regarding NF and PD does not include the utilization of the ILF method in particular. The promotion of automatic motor control by NF is believed to help patients (Sidhu and Cooke, 2021). Most reviews of PD in the NF literature utilize classical somatomotor rhythm (SMR)-based methods for NF that involve threshold-based training using a consciously mediated feedback (usually visual) reward. Cortical beta suppression with SMR training has been advocated on the observation that cortical beta oscillations are suppressed by levodopa (Doyle et al., 2005). Proposed mechanisms with SMR training include inducing plastic changes in the subthalamic nucleus (Fukuma et al., 2018), recruitment of unaffected nearby compensatory pathways (Philippens et al., 2017), and encouraging a shift toward more automatic motor control (Sidhu and Cooke, 2021).

### Neurofeedback Mechanisms

The STN is integral to the cortico-basal ganglia-thalamocortical complex and coherent beta oscillations have been demonstrated throughout this network in PD, especially between the STN and GPi (Brown et al., 2001), GPi and cortex (Williams et al., 2002), STN and thalamus (Hanson et al., 2012), and STN and cortex (Litvak et al., 2011).

Abnormal neuronal oscillations in STN in the beta frequency range are a characteristic observation in patients with PD, and treatment with levodopa attenuates the beta band power (Giannicola et al., 2010) while improving symptoms such as bradykinesia and rigidity.

In a recent study (Fukuma et al., 2018), EEG signals from bilateral inactive STN-DBS electrodes were recorded from 8 patients with PD during replacement of their pulse generator implants (using local anesthesia) as they rested on the surgical table 3 h after medication. Traditional neurofeedback (up-training and down-training, 4 patients in each group) was performed for 10 min using a 0–1 range scaled beta band power from pre-neurofeedback selected adjacent DBS contacts to provide the visual feedback (enlarging a circle scaled 0–1 with 1 being the largest diameter for those down-training, and vice versa (anti-correlated for those up-training). The training induced changes in the beta band power of the selected DBS contacts in the targeted direction for each group after 10 min; for all patients in the down-training group the beta band power was significantly decreased after the training. The powers in the other frequency bands were not changed significantly. There was also no apparent change in the patients' symptoms, especially *tremor* (EMG was also measured from muscles in both hands during the surgery). The investigators surmised the 10 min of feedback training might not be long enough; it is also possible the beta band power is not directly involved with causing tremor. Other investigators propose that the parkinsonian rest tremor probably has an independent pathophysiological substrate (Hammond et al., 2007).

Relatedly, stimulation of the posterior hypothalamus has been shown to restore locomotion in rats with haloperidol-induced akinesia (Young et al., 2011), probably by inducing frontal cortex slow delta (and concomitant beta-suppression) (Sano et al., 1970). Other NF investigators have recently proposed that training to decrease central alpha power at scalp sites over the supplementary motor area (SMA) results in activation of SMA to replicate activity patterns reflecting “autonomous locomotion,” and that this shift toward increased motor automaticity (requiring less conscious motor control) benefits whole body performance (Sidhu and Cooke, 2021).

To demonstrate the benefits of NF in experimental PD, 5 marmoset monkeys received 9–12 weeks of classical SMR training followed by MPTP injections to induce parkinsonian symptoms. They were compared to 5 marmoset monkeys who did not receive the training before and were also subjected to MPTP-induced PD. After disease stabilization all 10 monkeys were treated with levodopa (12.5 mg/kg PO BID for 3 weeks). All 10 monkeys were then euthanized for pathological examination. The investigators demonstrated that the monkeys who received SMR training had significantly reduced MPTP-induced PD symptoms (and reduced body weight loss) compared to controls, but there were no differences in pathological specimens in regard to cell loss in substantia nigra. Based on this neuropathological finding the authors concluded that no neuroprotective benefit was discernible and proposed instead that SMR training might enhance compensatory mechanisms (Philippens et al., 2017).

### Second Generation Neurofeedback

**Figure 1:**
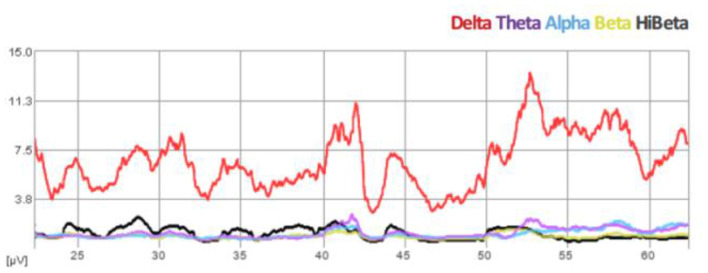
Spectral EEG demonstration of beta suppression during ILF brain training session (Case B).

We propose that infralow frequency EEG brain training (ILF NF) engages the brain's infraslow frequency networks that are non-neuronal in origin and exert extensive neuromodulatory effects on all neuronal populations, regardless of where they exist in the hierarchy of motor control in PD. We consider ILF a second-generation form of neurofeedback that involves limbic learning (Dobrushinaa et al., 2018); it directly engages with predominantly subcortical networks to encourage neuromodulation toward a renewed homeostasis and does not require the conscious effort required in traditional and classic neurofeedback methods. In this way ILF naturally promotes “automation” of motor systems. We consider the suppression of cortical beta (>12 Hz) and “high beta” (> 20 Hz) a standard expected result of the ILF method, evidenced by our real time trend graphs during every session (See Figure 1). Beneficial clinical effects (e.g., reduced tremors) are observed immediately following each 50-min session and sustain for up to 48–72 h. Repeat sessions are required for reinforcement and consolidation of learning to occur (Hellyer et al., 2016). In regard to potentially stimulating compensatory pathways, we prefer to consider instead that ILF brain training has a robust effect on neuroregulation at all levels in the hierarchy of motor control by potentiating adaptive neuroplasticity; we have discussed elsewhere its engagement with the slow control system in the brain including hypothalamic regulatory networks (Legarda et al., 2022).

### A Focus on the Mechanism of ILF Brain Training

A proposed mechanism for the ILF regime (Legarda et al., 2011), postulating that this form of neurotherapy engages directly with the brain's resting state networks in the awake (alert but non-engaged) state, appears to have stood the test of time. These relevant regulatory networks are non-neuronal in origin and are dependent on the ATP from astrocytes (Lorincz et al., 2009). Exemplified by the “task-negative” default mode network (DMN), resting state oscillations are associated with the primary assignments of brain function when we are awake but non-engaged: self-referential mental activity, correlation, adaptation, response generation, and homeostatic control—our subconscious vigilance and interoception (Buckner et al., 2008). In terms of neuroanatomic correlation, the hypothalamic-limbic system is integral to achieving this state of affairs. In terms of histologic-neurophysiologic correlation, the astroglial networks acting within their heterogenous neuronal domains are implicated.

In brief, ILF neuromodulation engages with the brain's infra-slow fluctuations, the widespread intrinsic networks that generate them, and impacts global neuro-regulation that includes hypothalamic-limbic circuitry. The hypothalamus in particular has been associated with the “slow regulation system” (Aladjalova, 1957, 1964). Regardless of where we train at the scalp the whole brain is involved in the program of optimizing self-regulation. Brain sites from which we record are cortical regions selected for being preferentially involved in the process (Hellyer et al., 2016). They are the multi-modal association areas, which are also hubs of the DMN, and they inform the salience network, which in its controlling role mediates shifts in dominance between the DMN and central executive networks (Sridharan et al., 2008). The ILF training is tailored to each individual with respect to both sensor placement and target frequency based on presenting symptoms and the neurological evaluation.

### Parkinson's Disease Brain Training Protocols

We share our experience of ILF neurotherapy for patients with PD by describing three patient profiles to illustrate our understanding of how this form of neurotherapy benefits individuals with PD.

Case A: 77-year-old female with uncontrollable tremors and extreme gait difficulty requires a walker for mobilization. After medical therapies are instituted, she is able to become independent of her walker, then of her cane, and enjoys tremendous improvement of her quality of life. Over a span of 8 years the tremors become uncontrollable; additional medical options give little relief; she is using her cane again. She agrees to neurofeedback. After the very first NF session, her tremors were objectively and subjectively much reduced. She completed 40 sessions. At this time, she is stabilized with the tremors and continues to come intermittently for her brain training sessions (at times forgetting her cane as she leaves!).

**Figure 2:**
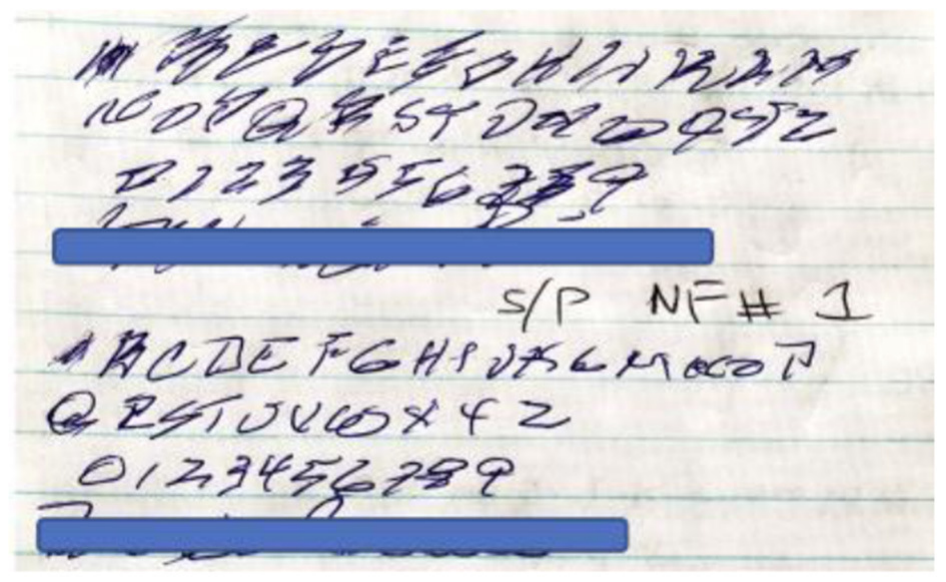
Case B demonstrates writing skills before (above) and after (below) her first session of ILF brain training.

Case B: 63-year-old college professor presents with voice difficulties and mild dysphagia, and on exam has no cogwheel rigidity, imbalance, or tremors. She responded well to speech dysphagia therapies and feels improved on carbidopa-levodopa monotherapy. Over a span of about 8 years, she develops tremors that are controlled by incrementally adding low dose pramipexole to 0.25 mg TID. She travels abroad for experimental therapy with fetal cell infusions and believes she derived further benefit. Over the next 2 years she begins to have writing difficulty and marked dysgraphia. She is requested to provide a writing sample. Immediately after a single neurofeedback session a repeat writing sample reveals improvement of the dysgraphia (Figure 2). She continued with the brain training and after 50 sessions feels well able to be independent in maintaining her own writing skills.

Case C: A 76-year-old lawyer presents with intracerebral hemorrhage on account of cerebral amyloid angiopathy. Six months later he develops elements of vascular parkinsonism. The patient is placed on PD medications with good response; however, over the span of 5 years he becomes immobile, unable to initiate motion, and totally dependent on wheelchair mobility. He sits for a session of neurofeedback; after a single session he is immediately able to start ambulating with a walker. He comes for repeat sessions to improve and sustain the learning effect over time.

*Training protocols*: Each brain training session lasts 50 min which limits the training to five sites at 10 min each. We begin at the basic training sites:

- T4-P4 (for parietal calming, regulation of sleep, and anxiety).
- T4-T3 (for instabilities; accessing amygdala-hippocampal complex and limbic system).
- T3-P3, T3-C3, and T3-F3 (for right hand dominant tremor).
- T4-P4, T4-C4, and T4-F4 (for left hand dominant tremor; to promote frontal-parietal connectivity and direct hand motor area regulation).
- Fp2+Fp1 fast (gamma, 40 Hz) synchrony: we later add bi-frontal fast synchrony training, believed to encourage microglial scavenging activity (of neurofibrillary tangles, amyloid plaques, etc.) (Martorell et al., 2019), and in doing so we remove the T3-F3/T4-F4 training sites.

This sequencing of training protocols may be modified for a PD patient needing to address other serious symptoms, for example dysphonia and dysphagia, wherein we would add T4-F8 and T3-F7 to include nearby opercular regions in the optimized self-regulation scheme. For problems with nausea, constipation, and autonomic instabilities we choose the T4-T3 placement. Impulse control behaviors are reduced by training at T3-Fp1. Rigidity, dyskinesias, and gait instability are notably reduced by training at the sites T4-P4 and T4-T3.

## Summary

Clinical benefits reported after performing traditional SMR-based neurofeedback in monkeys later injected with MPTP are compelling evidence for a learned resilience (Philippens et al., 2017). Neuromodulation in the ILF realm involves learning and remodeling by engaging with the neuroplasticity of slow oscillating networks that are non-neuronal in origin and that critically influence neuronal function, regulation, and functional integrity. Neuromodulation is actually training brain behavior (Othmer et al., 2013).

Progression of clinical PD as a neurodegenerative condition is not inevitable. There is growing evidence that astrocytic integrity is important to impede the progression of PD. We are proponents of the principle that there is a primary dysregulation involving specifically the astroglial network within the basal ganglia that leads to PD and ILF neuromodulation strengthens astroglial tasks that enhance dopaminergic neuronal integrity, thus slowing the progression of PD. We propose that ILF brain training is a non-invasive approach to re-regulate the brain's hierarchical motor system networks, forms part of an integrative approach in managing patients with medically refractory PD, and should be considered earlier before significant polypharmacy and before, or in anticipation of DBS. This form of neurotherapy requires some inconvenience on the part of the patient; it is a visit to the doctor's office once (ideally twice) a week, each session lasts 50 min, and they need to arrange for a consistent means of transportation.

## Future Directions

Abnormal beta oscillations of the STN and GPi are implicated in the pathophysiologic mechanism of PD and these regions are selectively targeted by DBS (Paff et al., 2020); however, chronic direct neuronal stimulation in the STN and GPi has been demonstrated to induce inflammation, local circuit remodeling, and chronic signal instability, resulting in reduced performance over time (Salatino et al., 2017, 2019), with the return or worsening of PD symptoms and comorbidities.

The origin of abnormal beta oscillations in PD remains unclear; investigators speculate that they are either the result of dysregulation of normal beta oscillations or are generated through entirely different circuit mechanisms (Li et al., 2014). Either way, their presence is associated with a motor system network in dyscontrol that is evidently remediable by way of clinically applied neuromodulation. A recent literature review concluded that clinical neurofeedback “appears to hold great potential as a treatment for PD motor symptoms” (Anil et al., 2021).

There is a multi-level regulatory hierarchy in the brain that is exemplified well by the motor system. Practitioners of ILF brain training empower patients to self-regulate via an evolutionarily established hierarchy of brain networks. All forms of neuromodulation have one prime objective: training brain behavior. The training proceeds most efficiently by targeting the regulatory hierarchy at its foundation through engagement with the brain's infralow frequencies, which takes us to the realm of the glia.

Engineering of surgically placed DBS devices is continually evolving; however, limitations on their long-term effectiveness still exist. Neuromodulation proceeds readily when no adversely permanent injury to local brain anatomy interferes with its prime objective. In our experience, incorporating ILF brain training-based intermittent neuromodulation early in the integrative management of disabling PD symptoms serves to postpone resorting to DBS, if not averting it entirely.

## Author Contributions

SL: 80% manuscript writing. DM: 5% manuscript writing and practice collaboration with ILF method. PM-M: 15% manuscript writing and practice collaboration with ILF method. All authors contributed to the article and approved the submitted version.

## Conflict of Interest

The authors declare that the clinical activity and literature review performed in preparation for this manuscript were conducted in the absence of any commercial or financial relationships that could be construed as a potential conflict of interest.

## Publisher's Note

All claims expressed in this article are solely those of the authors and do not necessarily represent those of their affiliated organizations, or those of the publisher, the editors and the reviewers. Any product that may be evaluated in this article, or claim that may be made by its manufacturer, is not guaranteed or endorsed by the publisher.
